# Experiential knowledge and peer support in type 1 diabetes: insights from endurance running communities

**DOI:** 10.1016/j.pecinn.2026.100500

**Published:** 2026-08-22

**Authors:** Jean-Charles Vauthier, Lucie Cholley, Paolo Di Patrizio, Patrick Mas, Delphine Arduini, Bernard Kabuth

**Affiliations:** a Université de Lorraine, France; b Département de médecine Générale, Faculté de médecine, Nancy, France; c InterPSY UR 4432, Université de Lorraine, Nancy, France; d Patient expert Association Française des Diabétiques, France; e Présidente association Type 1 Running Team, France

**Keywords:** Diabetes mellitus, type 1, Self-management, Social support, Peer group, Patient participation, Qualitative research, Physical endurance, Communities of practice

## Abstract

**Background:**

Managing type 1 diabetes (T1D) during endurance running requires highly individualized strategies, yet conventional healthcare offers limited sport-specific guidance. Peer communities have emerged as key sources of experiential knowledge, emotional support, and practical tools.

**Objective:**

To explore how endurance runners with T1D build self-management strategies, use peer networks and digital platforms, and integrate these exchanges into identity and daily life.

**Methods:**

Thirteen French-speaking adults with T1D who had completed at least one marathon or ultra-endurance event participated in semi-structured interviews. Data were analyzed using constructivist grounded theory. Two patient contributors living with T1D supported interpretation and writing. COREQ guidelines were followed.

**Results:**

Peer communities operated as informal educational systems that transformed individual trial-and-error into shared, actionable knowledge. Digital platforms provided rapid troubleshooting, reflective tools, and distributed mentorship. Role models facilitated confidence, self-efficacy, and identity reconstruction. Despite strong collective dynamics, management remained highly individualized through context-aware “micro-protocols” developed via safe experimentation and peer comparison.

**Conclusion:**

Peer communities play a central role in translating clinical recommendations into real-world endurance strategies. Integrating peer mentoring, digital literacy, and structured reflective practice into patient education may enhance personalization and confidence for people living with T1D.

**Innovation:**

This study demonstrates how communities of practice and digital infrastructures can support experiential learning in high-variability contexts and illustrates the contribution of patient partners to qualitative analysis and manuscript co-construction.

**Patient or public contribution:**

Two individuals living with T1D contributed to interpretation and writing, ensuring relevance and accessibility for the patient community.

## Highlights

• Peer communities function as informal educational systems for adults living with T1D engaging in endurance running.
• Digital platforms support rapid feedback, reflective practice, and distributed mentorship.
• Role models and peer visibility foster confidence, identity reconstruction, and resilience.
• Athletes develop individualized micro-protocols through safe, peer-supported experimentation.
• Implications for patient education include co-design with patient experts, digital literacy, and structured reflective tools.

## 1 Introduction

Over the past century, advances in insulin therapy and diabetes technologies have profoundly transformed the lives of people living with type 1 diabetes (T1D), making participation in demanding physical activities increasingly achievable [1], [2], [3]. While physical activity is widely recommended for people with T1D [4], [5], endurance sports— particularly marathon and ultra-endurance running—remain a complex challenge [6]. Fear of hypoglycemia, glycemic unpredictability, and precise insulin/nutrition adjustments create barriers to participation [7], [8], [9].

Despite these challenges, increasing numbers of people living with T1D now participate in endurance running, including marathons and ultra-trails [10]. Their participation highlights the need to better understand how individuals learn to manage diabetes in contexts characterized by uncertainty, physiological variability, and limited sport-specific guidance [5], [11]. Beyond clinical recommendations, these athletes often rely on experiential knowledge developed through trial and error, reflection on practice, and interaction with other runners living with T1D [12], [13], [14]. Most studies on exercise and T1D have focused on physiological responses, technological innovations, and clinical management strategies [5], [6], [10], [11], [15]. By contrast, the lived experience of endurance athletes with T1D has received limited qualitative attention. An exception is the work of Montt-Blanchard et al. (2022), which explored how marathon training contributes to the ongoing negotiation of diabetes in everyday life [14]. However, little is known about how experiential knowledge is collectively produced, shared, and refined within peer communities, and how these exchanges shape both self-management practices and athlete identities [13], [16]. This gap underscores the relevance of a qualitative approach centered on collective dynamics and patient expertise.

Managing T1D in the context of endurance sports requires an individualized, sport-specific approach [17]. This involves meticulous adjustments to insulin therapy and carbohydrate/nutritional strategies across exercise phases [11], [18]. While clinical guidelines offer general recommendations, many athletes refine these strategies through experiential learning and peer support. In this regard, patient communities may play an important role in bridging the gap between biomedical knowledge and the lived realities of endurance running with T1D [13], [19]. Beyond documenting practice, this study explores peer communities as informal educational systems that structure experiential learning, personalization, and safe experimentation in high-variability contexts [20]. Endurance running also offers a paradigmatic “extreme-variability” case, in which the challenges of self-management are intensified, making visible strategies and peer-mediated learning processes that may remain less apparent in routine contexts [10], [14].

This study explores the lived experience of endurance runners living with type 1 diabetes and the role of peer communities in shaping self-management practices and identity. Through a qualitative analysis of semi-structured interviews, we examine how these runners navigate the dual demands of chronic illness and prolonged physical exertion, and how experiential knowledge is developed, shared, and adapted within peer networks.

Drawing on concepts of experiential knowledge and patient expertise [12], together with notions of symbolic capital, resilience [14], [21], and collective identity [22], we seek to better understand how runners living with T1D construct, share, and mobilize knowledge in this highly demanding context. By bridging biomedical and sociological perspectives, this research contributes to a deeper understanding of experiential knowledge and patient involvement in healthcare.

In addition to its theoretical and clinical contributions, this study also aligns with the principles of patient and public involvement in healthcare research. By involving individuals living with T1D—not only as participants but also as contributors to the interpretation and writing process—it highlights the value of experiential knowledge and co-construction in health research and patient education. The nature and extent of this involvement are detailed in the Patient and Public Involvement section below.

## 2 Patient and public involvement

This study was informed by prior ethnographic engagement with endurance running communities of individuals living with type 1 diabetes. The principal investigator, himself a T1D runner, participated in races and informal peer encounters, which helped shape the research questions and interview guide.

During a national congress, preliminary findings were presented to a group of patients, including two individuals living with T1D who later became co-authors. Their feedback during this session contributed to refining the interpretation and framing of the results.

One of these contributors is a formally recognized patient expert, while the other is an active member of a patient association. Both participated in iterative discussions with the research team, helping to clarify key concepts and ensure the relevance and accessibility of the manuscript. Their involvement went beyond simple review, contributing to the co-construction of the narrative and analytical framing.

While their participation did not extend to the analysis of interview transcripts, in accordance with ethical commitments made to participants regarding data confidentiality and anonymity, their experiential insights were central to the interpretive process. The principal investigator maintained a reflexive stance throughout, documenting how his dual role influenced the integration of patient perspectives.

## 3 Methodology

### 3.1 Study design and theoretical orientation

We conducted a qualitative study based on semi-structured interviews, using a constructivist grounded theory (CGT) approach [23] to examine how peer communities contribute to self-management, experiential learning, and identity reconstruction among endurance runners living with type 1 diabetes (T1D). In line with Charmaz's constructivist perspective, CGT assumes that data and meanings are co-constructed through interactions between participants and researchers rather than discovered as objective realities. The approach combines iterative data collection and analysis, constant comparison, memo-writing, and progressive conceptual development while acknowledging the interpretive role of the researcher throughout the analytic process. This methodology was particularly well suited to exploring how experiential knowledge is generated, shared, and adapted within peer communities operating in a context of substantial physical and metabolic variability.

### 3.2 Setting and data collection period

Interviews were conducted via secure videoconference between July and December 2024, allowing participation from runners across different regions. All interviews were conducted in French, audio-recorded with consent, transcribed verbatim, anonymized, and deleted after transcription.

### 3.3 Participants and recruitment

Participants were eligible if they were adults aged 18 years or older, living with type 1 diabetes, French-speaking, and had completed at least one marathon or ultra-endurance event within the previous five years.

Participants were recruited using purposive sampling through personal contacts and closed online communities dedicated to T1D and endurance sports. These communities represent a small national population (<100 individuals according to community administrators and athlete estimates). Thirteen runners volunteered and completed an interview, a sample size consistent with the rarity of this population and adequate for grounded-theory saturation.

### 3.4 Sample size and saturation

We used a combination of purposive sampling and iterative analytic monitoring of saturation. After 13 interviews, no new conceptual categories emerged, and existing themes were consistently reinforced across transcripts. Recruitment was therefore closed based on thematic saturation rather than numerical targets.

### 3.5 Interview guide

The semi-structured interview guide explored the following domains:

– Personal and athletic trajectory; experience of living with T1D.

– Preparation routines before endurance events (insulin adjustments, nutrition planning, risk anticipation).

– Practices and decision-making during running events (monitoring, fueling, managing hypoglycemia/hyperglycemia, navigating uncertainty).

– Engagement with peer communities (online/offline), types of exchanges, perceived benefits and limits.

– Use of digital platforms for race reports, reflective writing, or sharing strategies.

– Emotional experience, identity, confidence, fears, and coping mechanisms.

– Interactions with healthcare professionals regarding sport and T1D.

– Contextual factors mentioned spontaneously (work–life organization, family, practical/financial accessibility).

– Final reflective insights or advice participants would give to other runners with T1D.

The guide was adapted iteratively as analysis progressed (e.g., questions on digital interactions were added after early interviews highlighted their importance). The initial guide is provided in Appendix A.

### 3.6 Researcher reflexivity

The principal investigator (PI) is a general practitioner and endurance runner living with T1D, which facilitated rapport and contextual understanding. To mitigate potential bias, the PI maintained a reflexive journal, documenting assumptions and interpretive decisions. All analytical steps were discussed with a multidisciplinary team (GPs, psychiatrist, psychologists, medical student) and two individuals living with T1D, allowing diverse perspectives to refine emerging interpretations.

### 3.7 Data analysis

Data were analyzed following constructivist grounded theory principles. All transcripts were imported into NVivo® (Release 1.7.2). Analysis involved: line-by-line initial coding; focused coding to refine categories; constant comparison across interviews; iterative memo-writing documenting emerging links between themes; and team discussions to refine the analytic structure.

Interpretations were reviewed with two contributors living with T1D to ensure resonance with experiential realities. We prioritized analytic depth, conceptual coherence, and credibility through triangulation, reflexivity, and transparent documentation of analytic decisions.

Reporting followed the COREQ 32-item checklist for interviews and focus groups; the completed checklist is provided as Supplementary Material [24].

### 3.8 Ethical considerations

The study received ethical approval from the South-East VI Research Ethics Committee (April 2024). All participants provided written informed consent. No financial compensation was offered. Identifying details were removed or altered in excerpts to ensure confidentiality.

## 4 Results

### 4.1 Participant characteristics

We interviewed 13 adults with T1D engaged in endurance running (11 men, 2 women). Median age was 44 years (range 26–66), with a median diabetes duration of 23 years [3], [4], [5], [6], [7], [8], [9], [10], [11], [12], [13], [14], [15], [16], [17], [18], [19], [20], [21], [22], [23], [24], [25], [26], [27], [28], [29], [30]. Most participants used insulin pumps (*n* = 9) and continuous glucose monitoring, predominantly Dexcom (*n* = 9, including one Dexcom G6), with others using Libre (*n* = 3) and Libre 2 (*n* = 1). The median longest distance completed was 59 km (42–200). Additional characteristics are summarized in Table 1.

**Table 1:** Characteristics of study participants.

ID	Sex	Age	Age at diagnosis	Duration of T1D	Treatment	CGM	HbA1c	Sports practiced	Max distance
S1	M	36	21	15	Pump (Omnipod)	Dexcom	7.8	Ultra-trail, ski, tennis-marathon	200 km
S2	M	26	15	11	MDI (pens)	Libre 2	N/A	Trail, triathlon, rugby-marathon	80 km
S3	M	53	38	15	Pump (Omnipod Dash)	Dexcom	N/A	Ultra-trail-marathon	108 km
S4	M	48	25	23	Pump (Omnipod, Diabeloop planned)	Dexcom G6	7.4	Trail, marathon	59 km
S5	F	49	23	27	Pump	Dexcom	5.8	Trail, ultra cycling, Marathon, triathlon	42 km
S6	M	41	19	22	Pump	Dexcom	N/A	Trail-marathon	50 km
S7	M	66	40	26	MDI (pens)	Libre	6.7	Trail-marathon	50 km
S8	M	37	13	24	Pump	Dexcom	N/A	Trail-marathon	60 km
S9	M	46	42	4	MDI (pens)	Libre	N/A	Trail-marathon	42 km
S10	M	38	10	28	Pump	Dexcom	6.4–6.5	Trail-marathon	100 km
S11	M	44	19	25	Pump	Dexcom	6–6.2	Trail-marathon	80 km
S12	M	26	23	3	MDI (pens)	Libre	N/A	Trail-marathon	42 km
S13	F	46	16	30	Pump	Dexcom	N/A	Trail-marathon	50 km

This table summarizes demographic and clinical characteristics of participants, including age, diabetes duration, treatment type, technology use, and athletic background. HbA1c values were not systematically collected; they are reported only when spontaneously mentioned by participants during interviews, in accordance with the protocol approved by the ethics committee.

### 4.2 Notes on quotations

All participant quotations below are translated from French interviews; participant IDs (S#) refer to the anonymized sample.

*Theme 1 - Community Formation and Shared Practices*

Participants described rare yet tightly knit communities of runners living with T1D that compensate for the lack of sport-specific advice in routine care and create a shared language connecting “runner” and “person living with T1D.” Several interviewees located the origin of these groups in a very practical need: “*we set up an association because there was no information on how to manage running with T1D… it lets everyone exchange about their practice and their way of handling diabetes—it's really facilitating*” (S1). These spaces curate practice-based tips (insulin tweaks, fueling, redundancy planning) and normalize trial-and-error learning, turning individual experiments into shared resources.

Beyond instrumental help, community participation reinforced belonging and collective identity. One runner spoke of moving from isolation to “*permanent exchange*,” sometimes anchored by a mentor with substantial ultra-distance experience: “*with the association it became a permanent exchange… and I had a real mentor who'd done several 160-km trails*” (S3). Others explicitly sought the collective to avoid being alone with problems: “*I was looking for that—not being alone… even just following others and seeing they have the same problems helped; there's only positive in these communities*” (S2). Across the corpus, runners also linked the community to resilience: normalizing setbacks, sharing troubleshooting routines, and reframing “*bad days*” as part of the craft.

Community practices were not limited to meetings or races; they extended to everyday pedagogy and public visibility. On long events, visible devices (pumps, CGM) or association jerseys naturally “*start conversations*,” offering occasions to explain and demystify diabetes—“*we do a bit of pedagogy while running*” (S1). Similar dynamics appeared in accounts from other participants (S5–S8, S10–S13), who described explaining their setup to fellow runners, reassuring curious spectators or parents, and using visibility to broaden assumptions about what is possible with T1D.

Importantly, the community does not erase individual variability; it helps people compare without prescribing. Several participants emphasized that they learned principles (anticipate, prepare backups, debrief, iterate) rather than templates, and that each athlete adapts to terrain, weather, duration, tech configuration, and personal thresholds. As one put it, “*what we all share… is that no one has the same approach*” (S1). Others (S7, S9–S12) highlighted how small, context-aware adjustments—co-designed with peers and later reviewed with clinicians—felt safer and more effective than one-size rules. In that sense, communities behave like informal educational systems that translate generic recommendations into actionable, safely testable micro-protocols while preserving autonomy.

Finally, communities carry a symbolic function. Participation, mutual aid, and shared narratives contribute to identity work—from the first marathon to longer ultras—by converting vulnerability (fear of hypos, uncertainty) into confidence grounded in preparation and collective know-how. This identity scaffolding was echoed across interviews (S1–S4, S6–S13) and frequently linked to paying it forward—supporting newcomers, reassuring families, and, for some, taking on an ambassador role within local clubs or public events.

*Theme 2 - Digital platforms as infrastructures for collective learning*

Digital platforms—blogs, private WhatsApp groups, Strava live-tracking, Facebook communities, and online race-report forums—formed a central architecture of peer learning for nearly every participant. Runners described these spaces as repositories of lived expertise, where hundreds of micro-tips (on sensors, fueling, basal adjustments, redundancy preparation, hydration, GI issues, altitude, night segments) converge into what one runner called “*a kind of collective intelligence*” (S3).

Several participants (S5–S8) emphasized how these groups offer crucial real-time troubleshooting, notably when facing adhesion problems, unexpected hypoglycemia, CGM calibration issues, or equipment breakdowns. One runner noted that in WhatsApp groups, answers appear “*in five minutes, even at night*,” transforming solitary doubts into supported decision-making (S7).

For others (S9–S12), digital communities were above all reassuring emotional environments. Seeing that others also struggled with CGM drift, digestive shutdown after several hours, or mood changes linked to glycemia helped normalize the experience: “*When I read that others hit the same wall, I stop feeling incompetent*” (S10).

Digital platforms also allowed distributed mentorship. Newcomers received guidance via screenshots, annotated curves, and context-specific tips (night segments, fasted runs, basal adjustments). One runner explained: “*I had no idea how to manage a 50 km. A guy from the group sent me his 3-page checklist. It saved my race*” (S12).

Still, participants warned against over-reliance on online advice. Many (S2, S4, S9) insisted on the need to “*triangulate information*” and honour individual variability:

“*What works for one diabetic can make another crash—it's comparison, not imitation*.” (S9).

Several explicitly criticized “*online templates*,” preferring flexible principles rather than prescriptive formulas.

Finally, digital platforms contributed to identity work and visibility. For S3, S5, S7, and S13, reading about other T1D athletes before attempting their own first marathon or ultra materially changed their sense of what was possible. As one put it:

“*Seeing others do it online made me believe it was doable before I even started*” (S3).

These exchanges helped transform isolation into belonging, and personal struggles into shared knowledge.

In sum, digital communities acted as infrastructures for collective learning, mixing technical expertise, rapid feedback, emotional support, and identity reinforcement, while preserving a shared ethic of individual adaptation.

*Theme 3 — Support, role models, and identity work*

Participants consistently described how peer support and relatable role models converted early fears (notably fear of hypoglycemia, equipment failure, or isolation) into confidence and planned exposure to challenge. For several newcomers, simply seeing other T1D runners complete long events online or in person was transformative: “*Seeing others do it online made me believe it was doable before I even started*” (S3). That recognition effect—“*I'm not alone, I'm not the only one facing this*”—was echoed across the corpus and framed as a first step toward action rather than hesitation.

Support took practical and emotional forms. Practically, peers shared how to prepare (micro-protocols, backups, what to carry and where), how to react to setbacks, and how to debrief after races. Emotionally, the group normalized doubt and helped place setbacks in a learning perspective. One participant summarized this shift succinctly: “*Seeing people who have the same problems as me… it helps so much*” (S2). Others described the community as a place where setbacks are reframed—not as failures, but as material for collective learning and next-time planning.

Role models were most effective when they were relatable rather than heroic. Runners valued examples that validated setbacks and disclosed the “*messy middle*” of learning (adhesion issues, GI shutdown, CGM lag at night), rather than only showcasing exceptional performances. One participant, active in an association, talked about “*pedagogy while running*”—conversations sparked by visible devices or jerseys during long events that allowed them to explain and demystify T1D to fellow runners and spectators (S1). This everyday visibility provided reassurance for peers and families: “you can see it in their eyes — they relax when they meet runners like us” (S11).

Identity work unfolded along two complementary lines. First, from vulnerability to agency: repeated, scaffolded experiences—backed by peers—helped participants reinterpret fear (hypos, night segments, remoteness) as a manageable variable within planned routines. As one runner put it, the community helped “*break the glass ceiling*” around what others imagine to be off-limits with T1D (S4). Second, from isolation to belonging: joining an association or a small online group turned a solitary craft into a shared project, where being a “*runner with T1D*” was not a label but a position from which to contribute, advise, and “*pay it forward*.” This was particularly salient among those who spoke with parents of newly diagnosed youngsters or who took informal mentoring roles for first marathons/ultras (reported across S5–S8, S10–S13).

Crucially, participants rejected the idea of the “*hero patient*.” They preferred horizontal support—“*comparison without prescription*”—and insisted that identity scaffolding works best when it embraces diversity: different speeds, distances, body types, devices, and risk tolerances. In practice, that meant celebrating progressions (first trail, first night section, first 50 km) as much as podiums; and narrating not only the wins but also the course corrections that made the next attempt safer and more enjoyable. This inclusive ethic—reported across interviews—helps explain why the same community could at once reassure a novice, challenge a seasoned runner to try a longer effort, and hold both when plans went sideways.

In sum, peer support and role models in these communities do more than motivate; they structure identity work. By normalizing fear, validating setbacks, and offering situated guidance, communities convert uncertainty into confidence grounded in preparation, while leaving room for plural trajectories rather than a single ideal of performance.

*Theme 4 — Plurality of strategies and individual autonomy*

Across interviews, runners repeatedly insisted that endurance with T1D has no single recipe. Communities helped them compare without prescribing, while each person built context-aware micro-protocols tuned to terrain, weather, event duration, device configuration, and personal thresholds. One participant put it bluntly: “*what we all share… is that no one has the same approach*” (S1). Rather than universal templates, what circulated were principles—anticipate, prepare backups, debrief, iterate—then adapt.

This plurality appeared in diverse insulin strategies, fueling preferences, and redundancy planning. For example, one runner described moving from halving basal and eating 60–80 g/h on long mountain trails to smaller pre-emptive basal reductions that reduced GI burden—“*it let me eat less and feel much better toward the end*” (S3). Another carried two spare pods and kept pens cool for long events, a self-devised tactic to preserve insulin efficacy and lighten mental load (S4). “*Several runners kept plan A/B/C, testing small and adjusting*” (S6).

Autonomy here did not mean isolation. Participants explicitly linked safe experimentation to scaffolded support: peers provided ranges, checklists, and caution points; runners then tested small changes and reviewed outcomes (CGM traces, sensations) before consolidating or discarding. Several (S7, S9–S12) emphasized boundary conditions in deciding when to override devices, increase fueling, or slow down. In practice, autonomy looked like dialogue: learn from others, try, track, and debrief—sometimes also with clinicians who valued the athlete's experiential data.

Plurality also reflected different relations to risk. Some prioritized robust setups, others lighter strategies based on early-warning cues. Community threads helped participants calibrate risk—for instance, when to trust trend arrows, when to revert to capillary checks if CGM drifted, or how to plan contingencies for adhesion failures deep into the race. This ‘situational autonomy’—shifting from device-led to body-led decisions—was repeatedly mentioned (S1–S4, S6–S13).

Finally, plurality and autonomy were tightly coupled to identity. For some, the craft of tuning micro-protocols was part of becoming “*a runner with T1D*” on their own terms; for others, limits were equally self-defined (e.g., opting for shorter formats or stepping back after a draining ultra). Communities validated diverse trajectories, celebrating firsts (first night section, first 50 km) as much as podiums, and treating course corrections as legitimate outcomes of learning rather than failures. This inclusive ethic helped autonomy remain safe, progressive, and sustainable.

In sum, the community's value was not to supply a prescriptive how-to, but to make personalization actionable: translating generic guidance into small, testable adjustments, cultivating backup habits, and legitimizing different risk/effort balances. Autonomy emerged not against community, but through it.

## 5 Discussion

### 5.1 Peer communities as informal educational systems

Across interviews, participants described communities that compensated for limited sport-specific advice in routine care and turned experiential knowledge into a usable, practice-based resource (e.g., pre-race insulin ranges, fueling under uncertainty), while validating emotional responses to risk and uncertainty. These observations support a plural model of expertise in which experiential authority complements clinical guidance without replacing it, echoing work on communities of practice in healthcare [13] and sociological perspectives on symbolic capital in sport [25]. In our data, “compare, don't copy” ethos (“comparison without prescription”) enabled runners to translate generic recommendations into context-aware micro-protocols co-constructed with peers and later refined with clinicians, directly informing patient education and counseling.

### 5.2 Digital platforms as infrastructures for collective learning and identity

Participants portrayed blogs, private WhatsApp groups, Strava live-tracking, Facebook communities, and race-report forums not merely as channels but as learning infrastructures that archive trials and errors, accelerate feedback. Runners posted questions and received near-real-time answers (including at night), transforming solitary doubts into supported decision-making; brief race debriefs turned individual experiments into shared capital for newcomers. These dynamics converge with prior analyses of peer conversations in virtual diabetes communities, where interactional patterns scaffold self-management learning [16]. They also echo practice-based accounts of how people domesticate illness through endurance practice and narrative—using reports and reflective texts to process experience and stabilize identity [14]. These observations are consistent with syntheses on the real-world use of automated and sensor-based systems, where gains in safety coexist with new forms of oversight and interpretation work [26], [27], [28].

Beyond technical troubleshooting (adhesion failures, CGM drift, fueling under GI distress), runners used platforms for reflective learning: documenting plans, outcomes, and “what I'd change next time,” which consolidated tacit know-how and facilitated distributed mentorship (e.g., annotated CGM curves, checklists, contingency lists). Short debriefs and annotated curves help operationalize personalization through small, testable adjustments [27], [29].

At the same time, participants expressed ambivalence toward highly curated “influencer” personas and prescriptive templates. They emphasized a “compare, don't copy” ethos: triangulate advice, situate it against one's own context (terrain, heat, night segments, tech configuration), and log outcomes before adoption. For counseling, this suggests two priorities: (i) teach digital literacy (source appraisal, limits of generalizability, signs of over-templating) and (ii) channel online activity toward reflective, safety-aware experimentation (brief debriefs; monitoring CGM trends; explicit boundary conditions). These priorities align with our data and complement existing calls to move from generic rules to principle-based personalization in exercise management for T1D [5], [10], while leveraging the specific affordances of virtual communities [16].

### 5.3 Support, role models, and identity reconstruction

Participants consistently described how peer support and relatable role models converted early fears (hypoglycemia, equipment failure, isolation) into confidence and planned exposure to challenge. Seeing other T1D runners complete long events—online or in person—reassured newcomers (“*seeing others do it made me believe it was doable before I even started*”) and reduced the sense of being alone with the disease, a pattern widely reported across our corpus. These effects were not merely motivational; they also supported concrete preparation and normalized doubt.

Endurance running provided a structured context to rehearse skills transferable to everyday management (planning, decisions under uncertainty, emotion regulation). This trajectory aligns with work on resilience in T1D, where protective processes emerge from repeated, supported exposure to manageable challenges, and where identity is strengthened by successful self-management episodes [21], [30]. Our findings also resonate with social identity perspectives highlighting how belonging to a valued in-group (here, runners with T1D) fosters self-efficacy while legitimizing diverse paths and paces [22]. Such identity-supporting processes are compatible with evidence that technology-enabled self-care can enhance confidence while demanding new literacies and routines [26], [27].

Crucially, participants rejected the “hero patient” ideal. Support worked best when it remained horizontal—a “compare, don't copy” ethos—validating setbacks (adhesion failures, CGM drift at night, GI shutdown) as material for collective learning rather than personal shortcomings. In practice, this meant celebrating progressions (first trail, first night section, first 50 km) as much as podiums and inviting runners to narrate course corrections that made subsequent attempts safer and more enjoyable. Such inclusive scaffolding turns vulnerability into confidence, without pressuring less-advanced athletes.

### 5.4 Plurality of strategies and autonomy: implications for individualized education

Participants rejected one-size templates and favored dialogical learning—observe, test, adapt, sometimes discard—underscoring the wide inter-individual variability of exercise management in T1D. In our data, runners combined different insulin tactics (e.g., modest pre-emptive basal reductions; temporary targets; keeping changes minimal), fueling logics (solid early → more liquid later; low vs. moderate carbohydrate intakes), and redundancy planning (spare pods/sets, tapes, fallback capillary checks), assembling context-aware micro-protocols tuned to terrain, heat, night segments, device configuration, and personal thresholds. This “compare, don't copy” ethos directly operationalized personalization and was routinely refined with clinicians when needed.

For patient education and counseling, these findings argue for structured personalization rather than prescriptive rules. Concretely, teams can co-create micro-protocols (e.g., pre-event insulin-reduction ranges, fueling windows, contingency plans), make boundary conditions explicit, and document outcomes (brief debriefs; CGM traces; “*what I'd change next time*”). This approach aligns with exercise guidance calling for individualized strategies in T1D and recognizes that device performance and carbohydrate needs shift with duration, intensity, and context [5], [10], [11]. Recent accounts of closed-loop use similarly argue for anticipatory tuning and context-aware overrides during prolonged aerobic efforts [26], [28].

Crucially, autonomy did not mean isolation. Participants linked safe experimentation to scaffolded support: peers offered ranges, checklists, and caution points; runners tested small changes and tracked results before consolidating or discarding; clinicians reviewed patterns when risks increased or problems persisted. This division of labour mirrors evidence that peer support improves self-management and that stakeholder engagement can surface patient preferences for peer mentoring within diabetes education [19], [31]. In practice, autonomy looked like dialogue across settings—community comparison, self-tracking and reflection, and clinic review—with each layer adding safety and context.

### 5.5 Implications for patient education and counseling

#### 5.5.1 Co-design with patient experts

Involve experienced T1D runners as co-facilitators of group sessions and co-authors of micro-protocols (e.g., pre-event insulin-reduction ranges, fueling windows, contingency lists). This improves relevance and uptake, and aligns with evidence that stakeholder engagement and peer support enhance self-management quality and acceptability [19], [31]. In our data, runners consistently described “comparison without prescription” as the engine of usable personalization, later refined with clinicians.

#### 5.5.2 Teach “safe experimentation”

Review CGM traces together to consolidate or discard small, logged adjustments. This operationalizes individualized exercise guidance and complements device-focused evidence on anticipatory adjustments [5], [18], [26]. Our participants spontaneously used this cycle (plan → do → review → adjust).

#### 5.5.3 Leverage digital reflective tools (race/activity debriefs)

Encourage short, structured debriefs—what I planned / what happened / what I'd change—and the use of screenshots/annotated curves; promote archiving of fixes so they become shared resources. Analyses of virtual diabetes communities show how peer conversations scaffold learning by making tacit strategies explicit [16], [27], [29]. Race reports and quick notes consistently stabilized know-how and identity.

#### 5.5.4 Promote horizontal peer mentoring

Pair novices with peers for pre-event planning (packing templates, redundancy checklists) and post-event debriefs (what worked/failed, escalation rules). Patients often prefer peer involvement, which supports self-management [19], [31]. In our interviews, distributed mentorship (e.g., sharing checklists, annotated CGM) shortened learning curves without imposing templates.

#### 5.5.5 Teach digital literacy and “compare, don't copy”

Help patients triangulate advice, recognize the limits of generalizability, and avoid rigid online templates; orient them toward principle-based adaptation (anticipate, backup, debrief) and context-aware logging before adoption. This guidance is consistent with calls to individualize exercise management [5] and with observed interactional patterns in virtual communities where learning occurs through negotiated, situated advice [16], [29]. Our participants explicitly requested support to sort credible content from curated “influencer” narratives.

#### 5.5.6 Use self-efficacy as a design target and a monitoring lens

Make self-efficacy an explicit outcome: map micro-skills (e.g., planning a night segment, deciding when to revert to capillary checks, packing redundancy) to practice opportunities (progressions from 10 km → trail → ultra) and reflective tracking. Instruments and syntheses in T1D highlight the centrality of self-efficacy for sustained self-management [32], while exercise guidance underlines the need to adapt carbohydrate/insulin strategies to context [5], [18]. Our findings show how small wins, validated by peers, grow confidence.

#### 5.5.7 Embed clinician oversight via clear escalation rules

Differentiate peer-informed micro-adjustments (e.g., modest pre-event insulin changes, fueling ranges) from higher-risk changes (e.g., large basal shifts, recurrent severe events, hypoglycemia unawareness) that warrant timely clinical review. Specify escalation thresholds and integrate patient-generated artifacts (checklists; annotated CGM) into consultations. This preserves safety while respecting experiential authority and mirrors how participants combined community comparison, self-tracking, and clinic review.

### 5.6 Relation to existing literature

Our findings complement biomedical and guidance-oriented literature on exercise in T1D by detailing how patients operationalize advice in real-world endurance contexts—translating generic rules into actionable personalization through dialogue and shared narratives [4], [5], [11], [18]. They also resonate with evidence that technology-assisted strategies around exertion require anticipatory adjustments and iterative tuning rather than static prescriptions [5], [18]. In parallel, our results align with analyses of communities of practice in healthcare learning, clarifying the mechanisms—mutual aid, iterative exchange, identity support—by which communities enable learning in high-variability environments [13]. Finally, the digital layer we observed (race reports, WhatsApp threads) converges with work on peer conversations in virtual diabetes communities that scaffold self-management by making tacit know-how explicit [16].

### 5.7 Methodological considerations

Two issues warrant brief reflection. First, this is a small, rare population (French-speaking endurance runners with T1D), with saturation reached after 13 in-depth interviews; our aim is analytic transferability via rich description rather than statistical generalization. Second, the PI's dual role (T1D runner) enhanced contextual understanding; reflexive journaling and team triangulation acted as safeguards so that interpretations were not solely shaped by the PI's experience—consistent with constructivist grounded theory and COREQ-aligned reporting [23], [33], [34], [35]. Together, these choices prioritize interpretive transparency and credibility while acknowledging contextual specificity.

### 5.8 Innovation

This work advances patient education by operationalizing communities of practice in a high-variability context (endurance with T1D) and specifying how digital platforms can be leveraged for reflective learning and identity support beyond conventional self-management frameworks. Concretely, we document mechanisms through which communities convert experiential knowledge into small, testable adjustments (micro-protocols), normalize setbacks as material for learning, and sustain a compare, don't copy ethos that protects against prescriptive norms [13], [14], [16]. Unlike traditional models, the study integrates patient contributors into interpretation and writing, enhancing relevance and accessibility for end-users.

## 6 Conclusion

Peer communities help endurance runners with T1D bridge the gap between clinical guidance and the lived demands of prolonged exertion. They provide technical tips, emotional validation, and identity resources while preserving individualized management. For patient education and counseling, the priority is not to prescribe community narratives but to harness them—co-design content, scaffold safe experimentation, and guide digital engagement—so that experiential knowledge becomes a reliable ally to clinical care.

## Individual contributions

Details of author contributions will be provided after peer review.

## Declaration of generative AI and AI-assisted technologies in the manuscript preparation process

During the preparation of this work, the authors used Microsoft Copilot in order to assist with language refinement and structural editing. After using this tool, the authors reviewed and edited the content as needed and take full responsibility for the content of the published article.

## CRediT authorship contribution statement

**Jean-Charles Vauthier:** Writing – original draft, Project administration, Methodology, Investigation, Formal analysis, Conceptualization. **Lucie Choley:** Writing, Formal analysis. **Paolo Di Patrizio:** Writing – review & editing, Supervision, Project administration, Methodology, Conceptualization. **Patrick Mas:** Writing – review & editing, Validation, Conceptualization. **Delphine Arduini:** Writing – review & editing, Validation, Conceptualization. **Bernard Kabuth:** Writing – review & editing, Validation, Supervision, Project administration, Methodology, Formal analysis, Data curation, Conceptualization.

## Ethics declaration

Written informed consent to take part in the study and to publish the article has been obtained from all participants or their legal representatives. The privacy rights of participants have been observed.

This study was performed in compliance with relevant laws, regulatory frameworks and guidelines where the research took place.

This study was conducted in accordance with The study complied with the Declaration of Helsinki and French regulations governing research involving human participants. Ethical approval was obtained from the South-East VI Research Ethics Committee (April 2024), and all participants provided written informed consent.

This study was approved by the South-East VI Research Ethics Committee.

(Approval No. 2024-A00063–44)

## Funding

This research received no specific grant from any funding agency in the public, commercial, or not-for-profit sectors.

## Declaration of competing interest

The authors declare no conflicts of interest related to this manuscript.

## Appendix A Supplementary data

Supplementary material 1: Interview guide

Supplementary material 2: COREQ checklist
