# Supplementary material for: Experiential knowledge and peer support in type 1 diabetes: insights from endurance running communities
Source: PEC Innov. 2026 Aug 22;9:100500. doi: 10.1016/j.pecinn.2026.100500 (PMC13544074; doi:10.1016/j.pecinn.2026.100500)
Supplement: Supplementary file 1 [file mmc1.docx]

Projet T1CourEUR – Guide d’entretien

Thèse de sciences-Ecole Doctorale BioSE

Jean Charles VAUTHIER

Laboratoire INTERPSY – Axe PRISME

Université de Lorraine

Version du 13 novembre 2023

# Préambule

Bonjour [prénom]

Je suis Jean-Charles, je réalise mon travail de thèse sur la question des coureurs diabétiques de type 1.

Comme il est d’usage dans les pelotons de course à pied, est-ce qu’on peut se tutoyer et s’appeler par nos prénoms ?

Comme nous avons pu échanger avant cette rencontre, l’objectif de cet échange est d’avoir un dialogue ouvert. Je souhaite explorer les ressorts motivationnels, les impacts sur le vécu au quotidien, des coureurs à pied qui vivent avec un diabète de type 1 et qui se sont déjà engagés sur des courses d’endurance, que nous définirons par tout ce qui est au-dessus du marathon, sur route ou sur sentier.

Es-tu OK sur l’objectif ?

J’utilise une méthode d’analyse qualitative dite en théorisation ancrée c’est-à-dire que je cherche à comprendre un phénomène (ici les coureurs d’endurance diabétiques de type 1) à partir des expériences en vraie vie, en partant du vécu des sujets. Je connais déjà partiellement ce phénomène puisque j’ai pu réaliser des observations par moi-même, mais je souhaite prolonger cette expérience par des entretiens individuels.

J’imagine que nous allons pouvoir échanger pendant peut être une heure au maximum. Si tu souhaites mettre fin plus tôt à l’entretien il n’y a aucun problème, tu es parfaitement libre. Après 1 heure, si l’un et l’autre souhaitons prolonger, il n’y a pas de problèmes non plus.

L’objectif est de se sentir bien tout au long de l’entretien. Nous pouvons faire des pauses. Tu peux demander à repréciser quelque chose que tu aurais déjà dit…

Nous allons essayer de conserver une ambiance assez courtoise, voire conviviale. Si nous sommes l’un et l’autre à l’aise, cela simplifiera les échanges.

Acceptes-tu que j’enregistre cet entretien pour faciliter la retranscription ultérieure ?

Je te rappelle que tout ce qui est dit sera anonymisé pour le travail de rédaction scientifique.

Est-ce que ce préambule appelle des questions, des besoins de précisions ?

# Je mets en route l’enregistrement :

Peux-tu, pour que je puisse identifier l’enregistrement, me rappeler les initiales de ton nom prénom, et ton âge :

Me confirmes-tu que tu as participé à une épreuve de course à pied d’une distance d’au moins 42 km au cours des 5 dernières années ?

Es-tu bien diabétique de type 1 : OUI /NON

Depuis quand ?

Acceptes-tu les conditions et les modalités de cette étude nommée T1Coureur ? OUI/NON

# Question qui débutera chaque entretien :

**Peux-tu me raconter comment tu as vécu ta dernière course ?**

Au fil de la conversation, idée de thématiques qui peuvent être abordées :

(En gras les thèmes de relance éventuelle à privilégier)

**A propos de la maladie**

**Comment s’est construite la stratégie de gestion du diabète en course ?**

**Impact de l’apprentissage fait via la course à pied sur le vécu au quotidien**

Plus facile de faire face dans les situations ordinaires quand on gère en extraordinaire ?

**Modification des relations à l’entourage**

Rapport avec ses médecins

🡪ta famille

🡪ton entourage (par exemple professionnel)

Expert de sa maladie ?

Moins besoin des médecins ?

**Quel est le rôle des médecins qui te suivent ?**

Comment qualifierais-tu le rôle de ton entourage dans tes projets ?

**Rapport au danger**

**Est-ce qu’il y a déjà eu des mises en garde sur un éventuel danger à ce genre de pratique ? Par qui ? Es-tu d’accord avec cela ?**

Comment ce danger supposé en course impacte le sentiment de fragilité ou de vulnérabilité qu’on prête parfois aux personnes malades dans la vie de tous les jours ?

**Identité :**

**Portes-tu un t-shirt avec des allusions directes ou indirect au diabète ?**

Portes-tu les couleurs du diabète quand tu cours ?

Est-ce que tu postes des photos sur les réseaux sociaux ?

Espère-tu que cela sera vu par d’autres diabétiques.

**Est-ce une fierté d’être diabétique ?**

**Tu considères-tu « malade » ?**

**Motivation à courir ?**

**Courrais-tu avant d’être malade ?**

Qu’est ce qui te pousse ?

**Quel est le plus grand défi dans ton cas ? (sous-entendu la course ou le diabète ?)**

**Comment te sens-tu à l’arrivée d’une épreuve ?**

**La communauté des diabétiques :**

**Rôle des autres diabétiques**

# Conclusions :

Merci [prénom]

Je te remercie pour cet échange très riche.

Est-ce qu’il y a quelque chose que tu voudrais ajouter, une réflexion ou une idée que nous n’aurions pas aborder, ou quelque-chose sur lequel tu voudrais insister ?

Acceptes-tu que je te recontacte si besoin ?

Le besoin, cela peut être de revenir sur quelque chose que tu aurais dit aujourd’hui et que je voudrais préciser pour ne pas mal interpréter un propos.

Ou alors pour te proposer une nouvelle rencontre, sous des modalités identiques ou peut être en groupe. Il arrive qu’on ait besoin d’un focus group, c’est-à-dire une forme de table ronde pour échanger à plusieurs et faire apparaitre des notions qui n’apparaissent pas dans des entretiens individuels.

Ou enfin pour me suggérer d’autres personnes à interroger si j’ai besoin d’avoir plus de sujets interrogés

Dans une de ces trois conditions acceptes-tu que je te recontacte ?

# Je mets fin à l’enregistrement

J’espère que cela a été, que tu as bien vécu cette expérience.

Je te remercie chaleureusement au nom de toute l’équipe de recherche qui m’entoure.

Bonne journée/soirée

## T1CourEUR Project – Interview Guide

**Doctoral thesis – BioSE Graduate School**
**Jean‑Charles VAUTHIER**
INTERPSY Laboratory – PRISME Axis
University of Lorraine
**Version: 13 November 2023**

### Preamble

Hello **[first name]**,
I’m Jean‑Charles. I’m conducting my doctoral research on runners living with type 1 diabetes (T1D).

As is common in running groups, **would you be comfortable if we use first names and “you” informally?**

As discussed before this meeting, the aim is to have an **open conversation**. I’d like to explore motivations and the day‑to‑day impact of living with T1D among runners who have already taken part in endurance events—defined here as any distance **beyond a marathon**, on road or trail. **Are you comfortable with this objective?**

Methodologically, I use a **qualitative grounded theory** approach, i.e., seeking to understand the phenomenon (T1D endurance runners) from **real‑life experience**. I already know the field through prior observations, but I want to extend this with individual interviews.

The conversation will last **about one hour**. You may stop at any time. We can also continue beyond one hour if **both** of us wish to do so. The priority is that you **feel comfortable** at every stage. We can pause whenever needed, and you can ask to rephrase or clarify anything at any time. Let’s keep a **courteous, even friendly** atmosphere—being at ease usually makes it easier to talk.

**Do you agree to audio‑record the interview** to facilitate accurate transcription?
Everything you share will be **anonymized** in any scientific outputs.
**Any questions before we start?**

(Start recording.)
For identification purposes only: could you please tell me **your initials** and **your age**?
Can you confirm that you have run **at least one race of 42 km or more** within the past five years?
Do you live with **type 1 diabetes**? **YES/NO** — Since when?
Do you **agree** to take part under the conditions of this study, named **T1CourEUR**? **YES/NO**

### Opening question (to start every interview)

**Could you tell me about your last endurance race—how you experienced it?**

From here, use prompts as relevant. Themes in bold are priority follow‑ups; adapt order and depth to the participant’s narrative.

## Thematic prompts (semi‑structured)

### **About diabetes / self‑management**

- **How did you build your race‑day diabetes management strategy?**
- **Impact of learning through running on daily life**:
  Has managing diabetes during “extreme” situations made it easier to handle “ordinary” situations in daily life?

### **Changes in relationships and support**

- Relationship with your **physicians/diabetologist**
- Your **family**
- Your **broader environment** (e.g., workplace)
- **Do you see yourself as an “expert” in your own condition?**
- Do you feel you **need your doctors less** than before?
- **How would you describe your physicians’ role in your projects?**
- **How would you describe your entourage’s role** in your projects?

### **Risk and danger**

- **Have you ever received warnings** about potential danger in doing this type of sport? **From whom?** Do you **agree** with those warnings?
- How does supposed **race‑related risk** affect the way people perceive **fragility or vulnerability** in everyday life with a chronic condition?

### **Identity**

- **Do you wear a T‑shirt** with direct or indirect references to diabetes?
- Do you **wear diabetes colors** when you run?
- Do you **post photos** on social media?
- Do you **hope** other people with diabetes will see them?
- **Is it a source of pride to be diabetic?**
- **Do you consider yourself “ill/sick”?** (If relevant, explore nuances.)

### **Motivations for running**

- **Did you run before your diagnosis?**
- What **drives** you to run now?
- **What is the bigger challenge for you—the race or diabetes?**
- **How do you feel at the finish line** of an event?

### **The community of people with diabetes**

- **Role of other people with T1D** in your journey (peer support, tips, mentoring, groups, events, online communities).

## Closing

Thank you, **[first name]**.
Is there **anything you’d like to add**—a reflection or idea we didn’t cover, or something you’d like to emphasize?
**May I contact you again** if needed—for example:

- to clarify a point so I don’t misinterpret it,
- to invite you to another interview under similar conditions, or possibly to a **focus group** (round‑table discussion) to explore themes that may not surface in one‑to‑one interviews,
- or to **suggest other potential participants** if we need to broaden the sample.
  **Do you agree to be re‑contacted under any of these conditions?**

(Stop recording.)
I hope the experience was positive for you.
Thank you very much on behalf of the research team.
**Have a great day/evening.**
