# Supplementary material for: Experiential knowledge and peer support in type 1 diabetes: insights from endurance running communities
Source: PEC Innov. 2026 Aug 22;9:100500. doi: 10.1016/j.pecinn.2026.100500 (PMC13544074; doi:10.1016/j.pecinn.2026.100500)
Supplement: Supplementary file 2 [file mmc2.docx]

COREQ Checklist (Consolidated Criteria for Reporting Qualitative Research)

Domain 1: Research team and reflexivity

1. Interviewer/facilitator: Yes, specified (principal investigator).

2. Credentials: General practitioner, university lecturer-researcher, thesis supervisor in medicine.

3. Occupation: Academic general practitioner at the time of the study.

4. Gender: Male.

5. Experience and training: Experienced in qualitative research, trained in Grounded Theory and reflexivity.

6. Relationship established: Yes, prior immersion in peer support groups.

7. Participant knowledge of the interviewer: Yes, identity and background disclosed.

8. Interviewer characteristics: Reflexive posture, personal experience with type 1 diabetes and ultra-endurance sport.

Domain 2: Study design

9. Methodological orientation and theory: Constructivist Grounded Theory (Charmaz).

10. Sampling: Purposive sampling with iterative, theory‑informed adjustments; recruitment closed at thematic saturation.

11. Method of approach: Social media, word-of-mouth, peer networks.

12. Sample size: 13 participants.

13. Non-participation: None reported.

14. Setting of data collection: Videoconference.

15. Presence of non-participants: No.

16. Description of sample: Adults with type 1 diabetes, French-speaking, engaged in endurance sports.

17. Interview guide: Yes, enriched progressively.

18. Repeat interviews: No.

19. Audio/visual recording: Audio only, destroyed after transcription.

20. Field notes: Yes, research journal maintained.

21. Duration: Average of 57 minutes (range 47–68 minutes).

22. Data saturation: Thematic saturation reached after 13 interviews; no new conceptual categories emerged (see Methods).

23. Transcripts returned: No.

Domain 3: Analysis and findings

24. Number of data coders: Collaborative analysis with triangulation.

25. Description of the coding tree: Inductive coding, axial coding, categorization, theorization.

26. Derivation of themes: Yes, thematic structuring.

27. Software: NVivo®.

28. Participant checking: No formal member‑checking of transcripts; two T1D contributors (PPI) provided interpretive feedback on preliminary analyses and co‑wrote sections (see PPI & Data analysis).

29. Quotations presented: Yes, anonymized excerpts.

30. Data and findings consistent: Strong consistency, well-argued.

31. Clarity of major themes: Yes.

32. Clarity of minor themes: Yes, diversity acknowledged despite sociological homogeneity.

| **COREQ item(s)** | **What the item covers** | **Where reported in the PEC manuscript** |
| --- | --- | --- |
| 1–5 | Interviewer/facilitator; credentials; occupation; gender; experience & training | Methodology → Researcher reflexivity (PI is a GP, male, T1D runner; qualitative/CGT training; reflexive posture). |
| 6–8 | Prior relationship; what participants knew; interviewer characteristics | Patient and Public Involvement (prior immersion in T1D endurance communities; identity disclosed) + Methodology → Researcher reflexivity (dual role, influence addressed). |
| 9 | Methodological orientation (e.g., CGT/Charmaz) | Methodology → Study design and theoretical orientation (constructivist grounded theory, Charmaz). |
| 10–12 | Sampling strategy; approach to participants | Methodology → Participants and recruitment (purposive sampling via closed online communities/personal networks; iterative analytic monitoring of saturation). |
| 13 | Non‑participation (refusals/drop‑outs) | Not explicitly reported in the text; Checklist states none reported. |
| 14 | Setting of data collection | Methodology → Setting and data collection period (secure videoconference). |
| 15 | Presence of non‑participants | Not mentioned in the manuscript; Checklist notes No. |
| 16 | Sample description | Results → Participant characteristics + Table 1 (age, duration, therapy, CGM, sports; median age/duration). |
| 17 | Interview guide | Methodology → Interview guide (domains listed; Appendix A referenced). |
| 18 | Repeat interviews | Not performed; Checklist indicates No. |
| 19 | Audio/visual recording | Methodology → Setting and data collection period (audio‑recorded with consent; deleted after transcription). |
| 20 | Field notes | Methodology → Data analysis (memo‑writing; analytic notes) and Researcher reflexivity (reflexive journal). |
| 21 | Duration of interviews | Table 1 / dataset description: ~57 min on average (range 47–68 min). |
| 22 | Data saturation | Methodology → Sample size and saturation (thematic saturation after 13 interviews; no new categories). |
| 23 | Transcripts returned to participants | Not done; Checklist indicates No. |
| 24 | Number of data coders | Methodology → Data analysis (team discussions; collaborative coding/triangulation). |
| 25 | Description of coding tree | Methodology → Data analysis (line‑by‑line, focused coding; constant comparison; category refinement). |
| 26 | Derivation of themes | Methodology → Data analysis and Results (Themes 1–4; theory‑informed, inductive development). |
| 27 | Software | Methodology → Data analysis (NVivo® Release 1.7.2). |
| 28 | Participant checking (findings) | No formal member‑checking; two T1D contributors provided interpretive feedback and co‑wrote sections (PPI). Report this nuance in COREQ (e.g., “No formal transcript return; interpretive sense‑checking by two PPI contributors”). PPI + Data analysis. |
| 29 | Quotations presented | Results → integrated verbatim quotes with participant IDs (S1–S13); Notes on quotations specifies translation. |
| 30 | Data–findings consistency | Evidenced across Results ↔ Discussion (claims trace to data; e.g., micro‑protocols, debriefs, mentoring). |
| 31–32 | Clarity of major/minor themes | Results (four clearly delineated themes) and Discussion (matching sub‑sections); minor variations acknowledged (risk relations, trajectories). |
